# Supplementary material for: The MURAL collection of prostate cancer patient-derived xenografts enables discovery through preclinical models of uro-oncology
Source: Nat Commun. 2021 Aug 19;12:5049. doi: 10.1038/s41467-021-25175-5 (PMC8376965; doi:10.1038/s41467-021-25175-5)
Supplement: Supplementary file 1 — Supplementary Information [file 41467_2021_25175_MOESM1_ESM.pdf]

Supplementary Materials

- Supplementary Fig. 1: PDX take rates based on the source of tissue
- Supplementary Fig. 2: Enrichment of core signalling pathways in PDXs and generation of organoids from PDXs
- Supplementary Fig. 3: Genomic alterations in MURAL PDX cohort
- Supplementary Fig. 4: Clinical history of patients following tumor collection for research-ready PDXs
- Supplementary Fig. 5: Characterisation of research-ready PDX collection
- Supplementary Fig. 6: Individual data for PCTs and volcano plots per patient
- Supplementary Fig. 7: Pathology assessment of original tissues specimens and PDXs

Supplementary Figures

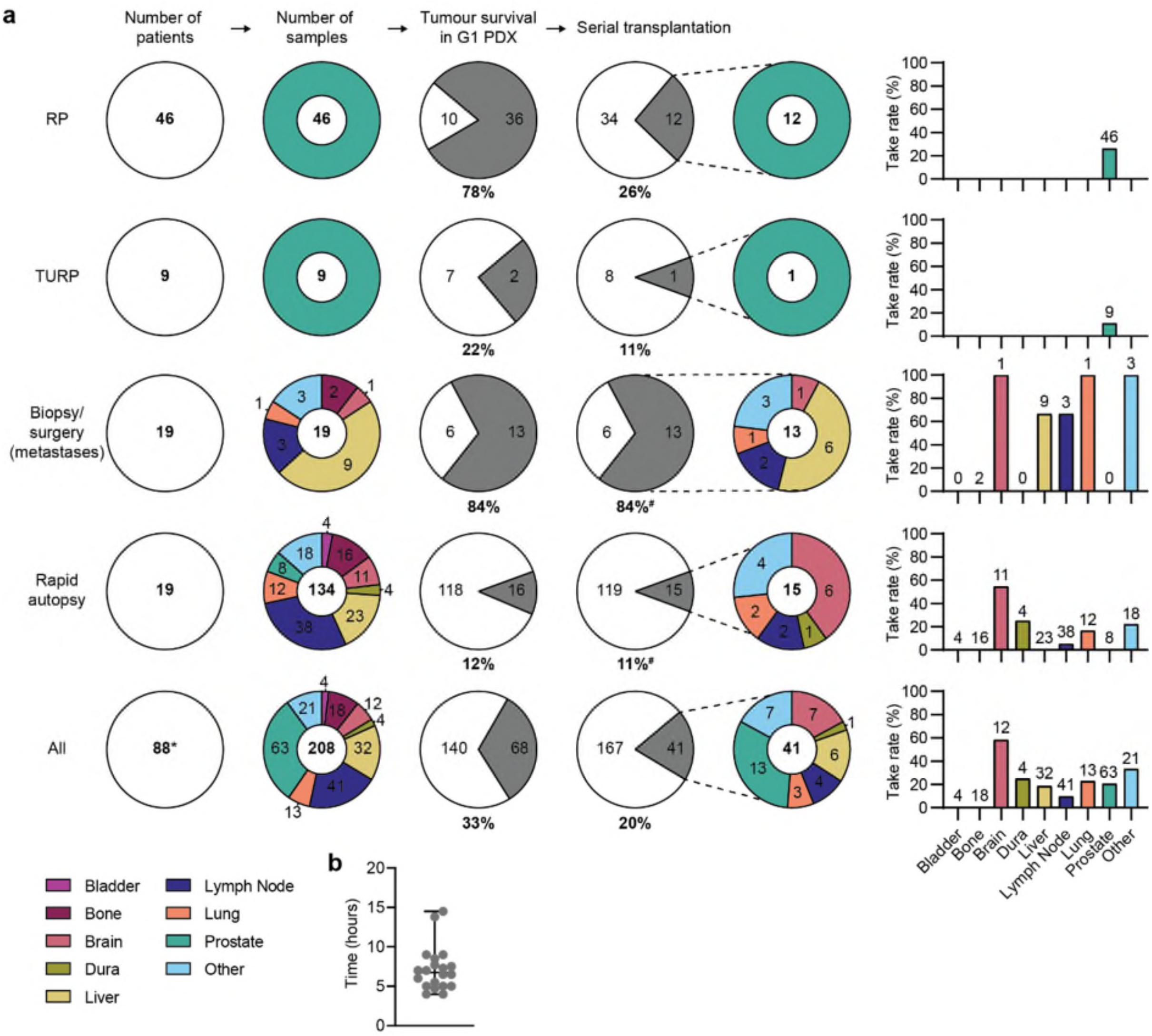

Supplementary Fig. 1: PDX take rates based on the source of tissue. (a) Pie charts show the numbers of patients who consented to donate tissue and the numbers of samples obtained from radical prostatectomy (RP), transurethral resection of the prostate (TURP), biopsies or surgery of metastases, and rapid autopsy (*For 5 patients, tissue was obtained from multiple sources, e.g. RP and surgical resection of a metastasis from the same patient). Colors denote the site that each sample was obtained from. The bar charts show the percentage take rates for each tumor site and tissue source, with numbers indicating the number of samples that were collected. #Among sample sources, there was a significant difference in the number of

tumors that grew as serially transplantable PDXs for biopsy/surgery samples of metastases and rapid autopsy specimens ($P < 0.0001$; two-sided chi-squared test with Fisher's exact approach for post-hoc analysis). (**b**) Plot showing the average time (hours) from death to the start of autopsy for patients who consented to the CASCADE rapid autopsy program ($n = 20$ patients). Data shown as mean ± SEM. Source data are provided as a Source Data file.

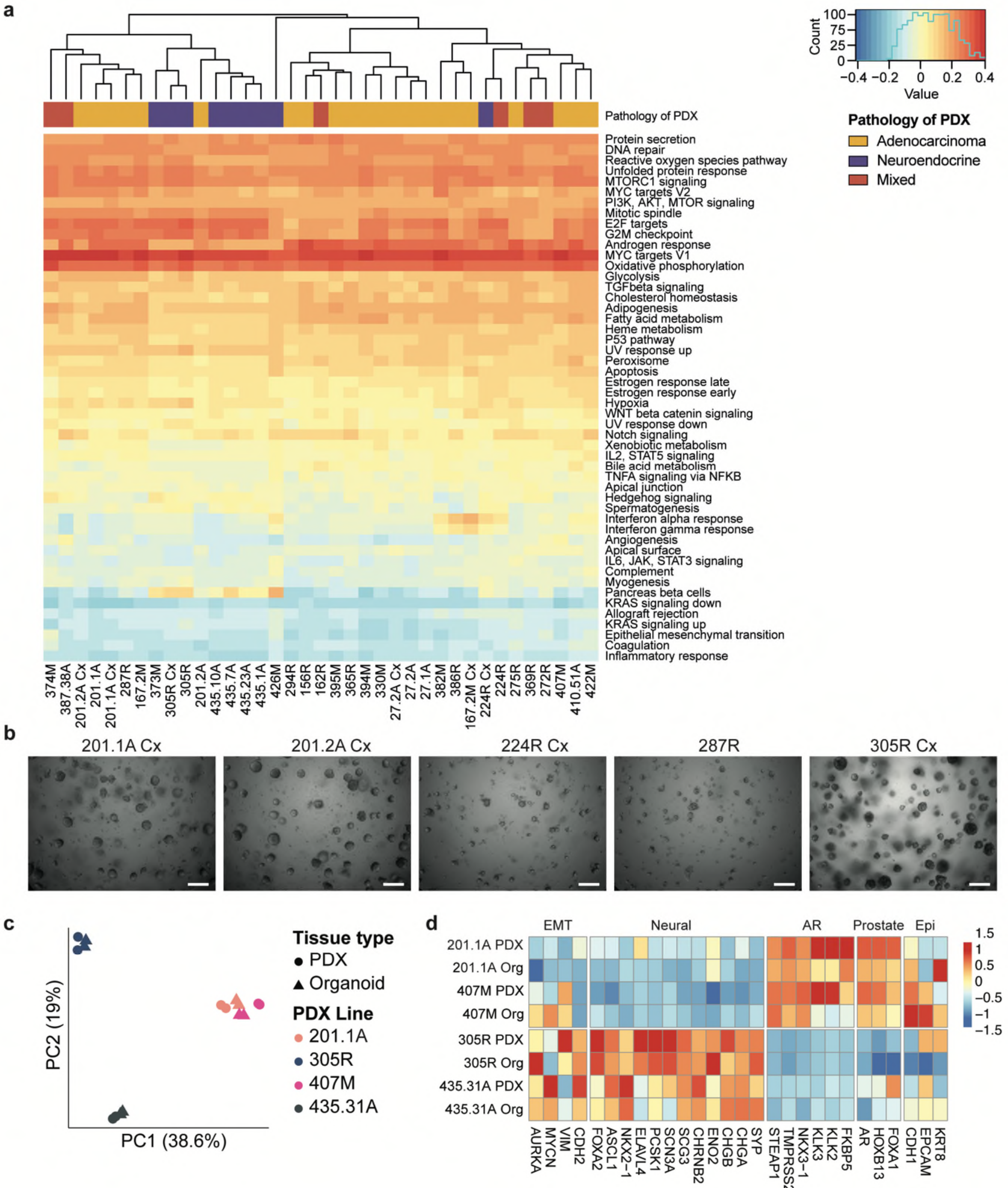
a
Count
100
75
25
0
−0.4 −0.2 0 0.2 0.4
Value
Pathology of PDX
Pathology of PDX
Adenocarcinoma
Neuroendocrine
Mixed
Protein secretion
DNA repair
Reactive oxygen species pathway
Unfolded protein response
MTORC1 signaling
MYC targets V2
PI3K, AKT, MTOR signaling
Mitotic spindle
E2F targets
G2M checkpoint
Androgen response
MYC targets V1
Oxidative phosphorylation
Glycolysis
TGFbeta signaling
Cholesterol homeostasis
Adipogenesis
Fatty acid metabolism
Heme metabolism
P53 pathway
UV response up
Peroxisome
Apoptosis
Estrogen response late
Estrogen response early
Hypoxia
WNT beta catenin signaling
UV response down
Notch signaling
Xenobiotic metabolism
IL2, STAT5 signaling
Bile acid metabolism
TNFA signaling via NFKB
Apical junction
Hedgehog signaling
Spermatogenesis
Interferon alpha response
Interferon gamma response
Angiogenesis
Apical surface
IL6, JAK, STAT3 signaling
Complement
Myogenesis
Pancreas beta cells
KRAS signaling down
Allograft rejection
KRAS signaling up
Epithelial mesenchymal transition
Coagulation
Inflammatory response
374M
387.38A
201.2A Cx
201.1A
201.1A Cx
287R
167.2M
373M
305R Cx
305R
201.2A
435.10A
435.7A
435.23A
435.1A
426M
294R
156R
162R
395M
365R
394M
330M
27.2A Cx
27.2A
27.1A
382M
386R
167.2M Cx
224R Cx
224R
275R
369R
272R
407M
410.51A
422M
b
201.1A Cx
201.2A Cx
224R Cx
287R
305R Cx
c
Tissue type
PDX
Organoid
PDX Line
201.1A
305R
407M
435.31A
PC2 (19%)
PC1 (38.6%)
d
EMT
Neural
AR
Prostate
Epi
201.1A PDX
201.1A Org
407M PDX
407M Org
305R PDX
305R Org
435.31A PDX
435.31A Org
AURKA
MYCN
VIM
CDH2
FOXA2
ASCL1
NKX2−1
ELAVL4
PCSK1
SCN3A
SCG3
CHRNB2
ENO2
CHGB
CHGA
SYP
STEAP1
TMPRSS2
NKX3−1
KLK3
KLK2
FKBP5
AR
HOXB13
FOXA1
CDH1
EPCAM
KRT8
1.5
1
0.5
0
−0.5
−1
−1.5

Supplementary Fig. 2: Enrichment of core signalling pathways in PDXs and generation of organoids from PDXs. (**a**) Heatmap and hierarchical clustering of single-sample gene set enrichment analyses (ssGSEA) for the 50 MSigDB hallmark gene sets based on RNA sequencing data for 39 PDXs in testosterone-supplemented and castrate (Cx) mice. The enrichment of each gene set is shown per sample, from low (blue), moderate (yellow) to high (red). Gene sets are hierarchically clustered using ssGSEA scores. Pathology of each PDX is shown, with adenocarcinoma in yellow, neuroendocrine in purple and mixed pathology in orange. (**b**) Representative brightfield images of five actively growing organoids established from PDXs. Scale bar = 500 μm, organoid establishment has been conducted independently at least 3 times for each PDX line (**c**) PCA plot of gene expression from RNA sequencing based on PC1 and PC2 for PDXs (circles) and organoids (triangles) for four PDXs lines. (**e**) Heatmap showing the relative gene expression between PDX and organoids (Org) for markers of epithelial to mesenchyme transition (EMT), Neural lineage, Androgen response (AR), prostate lineage and epithelial lineage (Epi)[1].

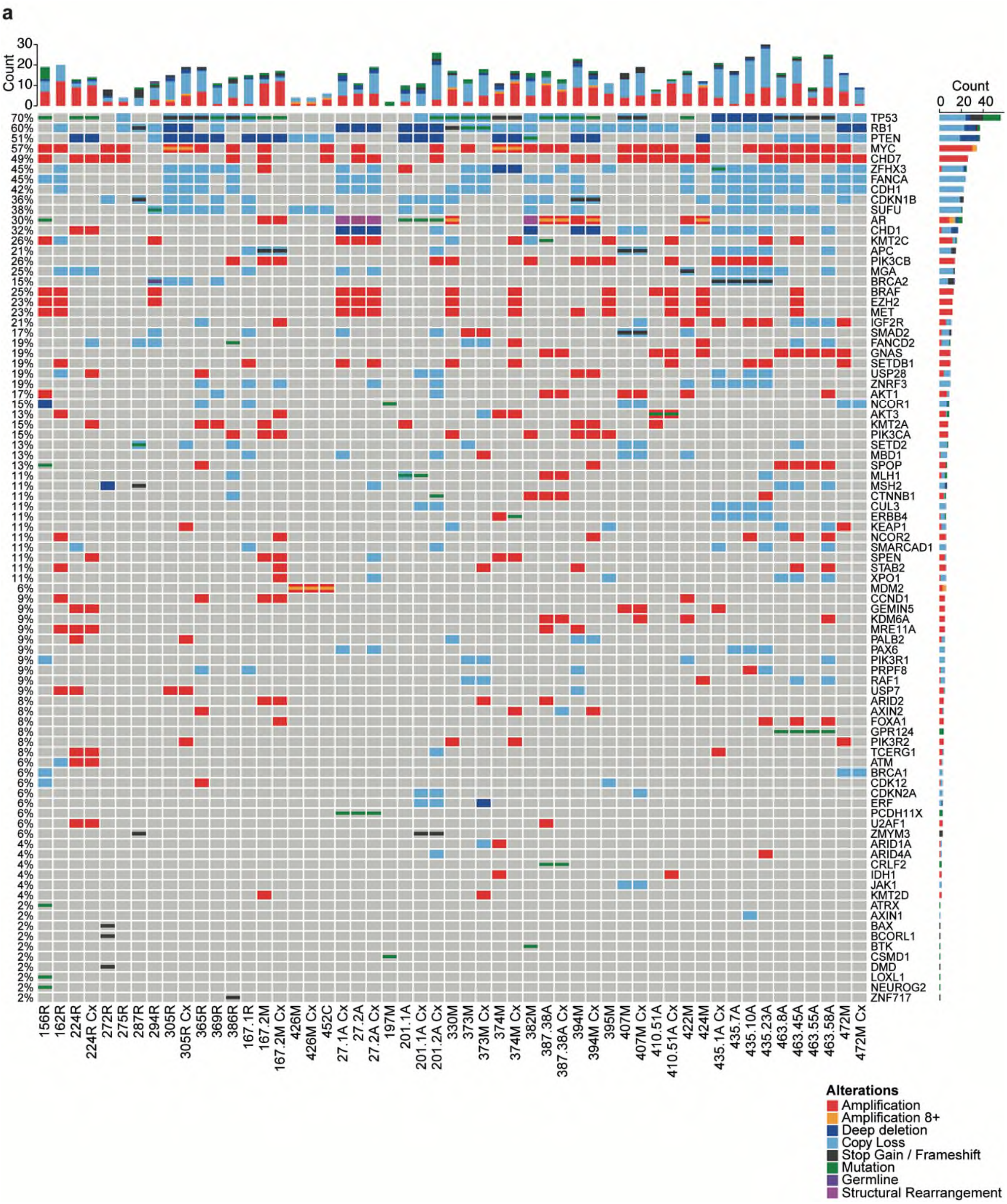
a
Count
Alterations
Amplification
Amplification 8+
Deep deletion
Copy Loss
Stop Gain / Frameshift
Mutation
Germline
Structural Rearrangement

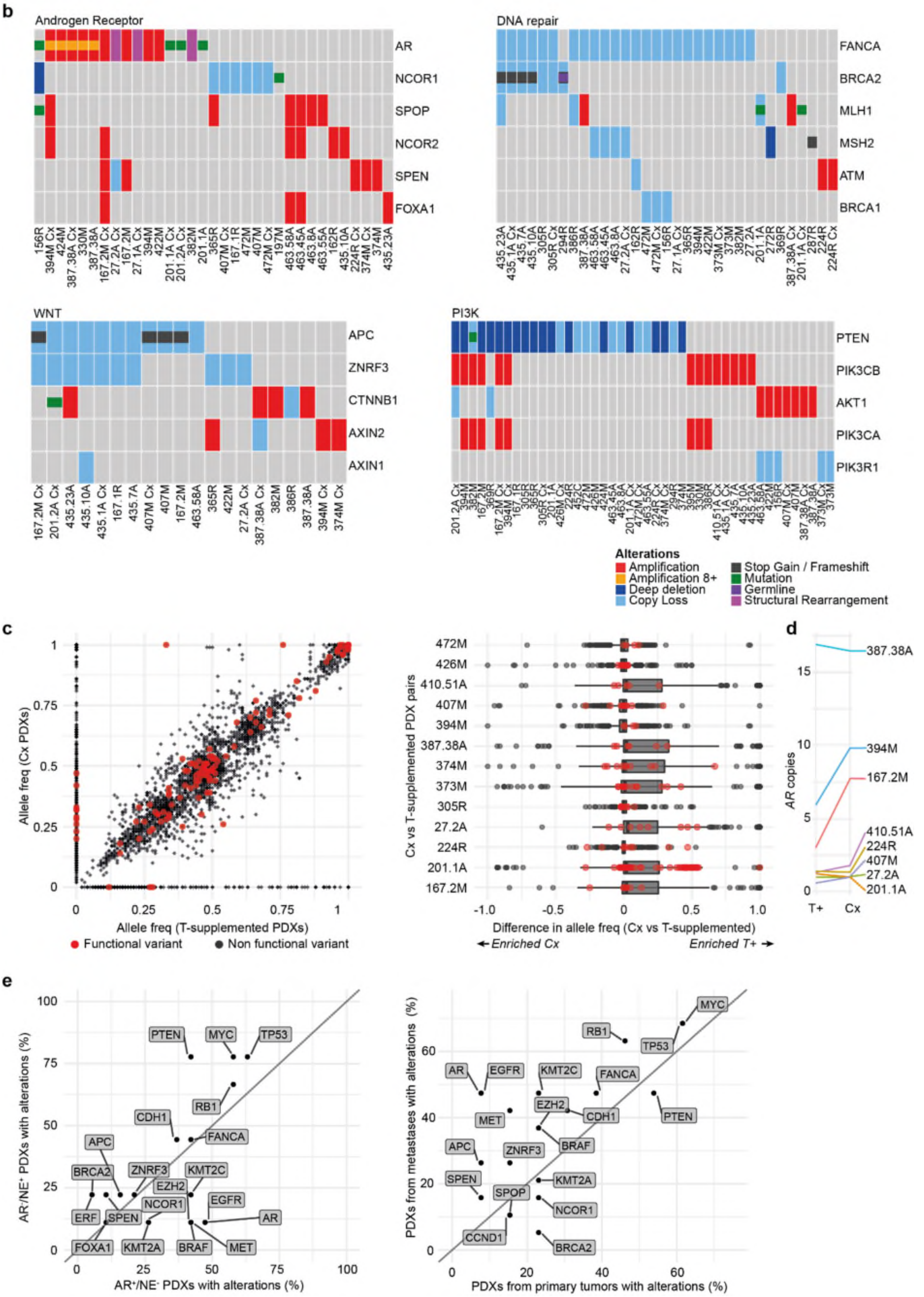
b
Androgen Receptor
AR
NCOR1
SPOP
NCOR2
SPEN
FOXA1
DNA repair
FANCA
BRCA2
MLH1
MSH2
ATM
BRCA1
WNT
APC
ZNRF3
CTNNB1
AXIN2
AXIN1
PI3K
PTEN
PIK3CB
AKT1
PIK3CA
PIK3R1
Alterations
Amplification
Amplification 8+
Deep deletion
Copy Loss
Stop Gain / Frameshift
Mutation
Germline
Structural Rearrangement
c
Allele freq (Cx PDXs)
Allele freq (T-supplemented PDXs)
Functional variant
Non functional variant
Cx vs T-supplemented PDX pairs
472M
426M
410.51A
407M
394M
387.38A
374M
373M
305R
27.2A
224R
201.1A
167.2M
Difference in allele freq (Cx vs T-supplemented)
Enriched Cx
Enriched T+
d
AR copies
387.38A
394M
167.2M
410.51A
224R
407M
27.2A
201.1A
T+
Cx
e
AR+/NE+ PDXs with alterations (%)
AR+/NE- PDXs with alterations (%)
PTEN
MYC
TP53
RB1
CDH1
FANCA
APC
BRCA2
ZNRF3
KMT2C
EZH2
EGFR
NCOR1
ERF
SPEN
AR
FOXA1
KMT2A
BRAF
MET
PDXs from metastases with alterations (%)
PDXs from primary tumors with alterations (%)
CCND1
SPOP

Supplementary Fig. 3: Genomic alterations in MURAL PDX cohort. (**a-b**) Genomic alterations in MURAL PDXs based on targeted DNA sequencing. Somatic nucleotide variations with 0.75 or greater allelic frequency are reported (amplification with 3 or more copies – red; amplification with 8 or more copies – yellow; deep deletion – dark blue; copy loss – light blue; stop gains and frameshift mutations – black; missense mutation – green; germline mutations – purple; structural rearrangements - pink). (**a**) Summary of genomic alterations that have previously reported in patient cohorts[2,3] that occur in MURAL PDXs. The percentage of PDXs across the cohort with one or more alteration/s per gene is shown to the left. The bar plot to the right shows the number of alterations per gene observed across the PDX cohort. The bar plot at the top shows the number of alterations observed in each PDX. Genomic structural rearrangements of the *AR* were reported previously[4]. (**b**) Heatmaps showing alterations in the AR, DNA damage repair, Wnt, and PI3 Kinase pathways. (**c**) Scatter plot and boxplot of allele frequencies (allele freq) in matching samples from earlier generation PDXs grown in testosterone-supplemented mice compared to later generation PDXs grown in castrated (Cx) mice. Red dots show functional variants, black dots show non-functional variants. (**d**) Plot *AR* copy numbers in matching PDXs from testosterone supplemented (T+) versus castrated mice. (**e**) Scatterplots showing the percentage of PDXs with any alteration in candidate genes (left: AR^-/NE^+ PDXs versus AR^+/NE^- PDXs; right: PDXs from metastases versus PDXs from primary tumors).

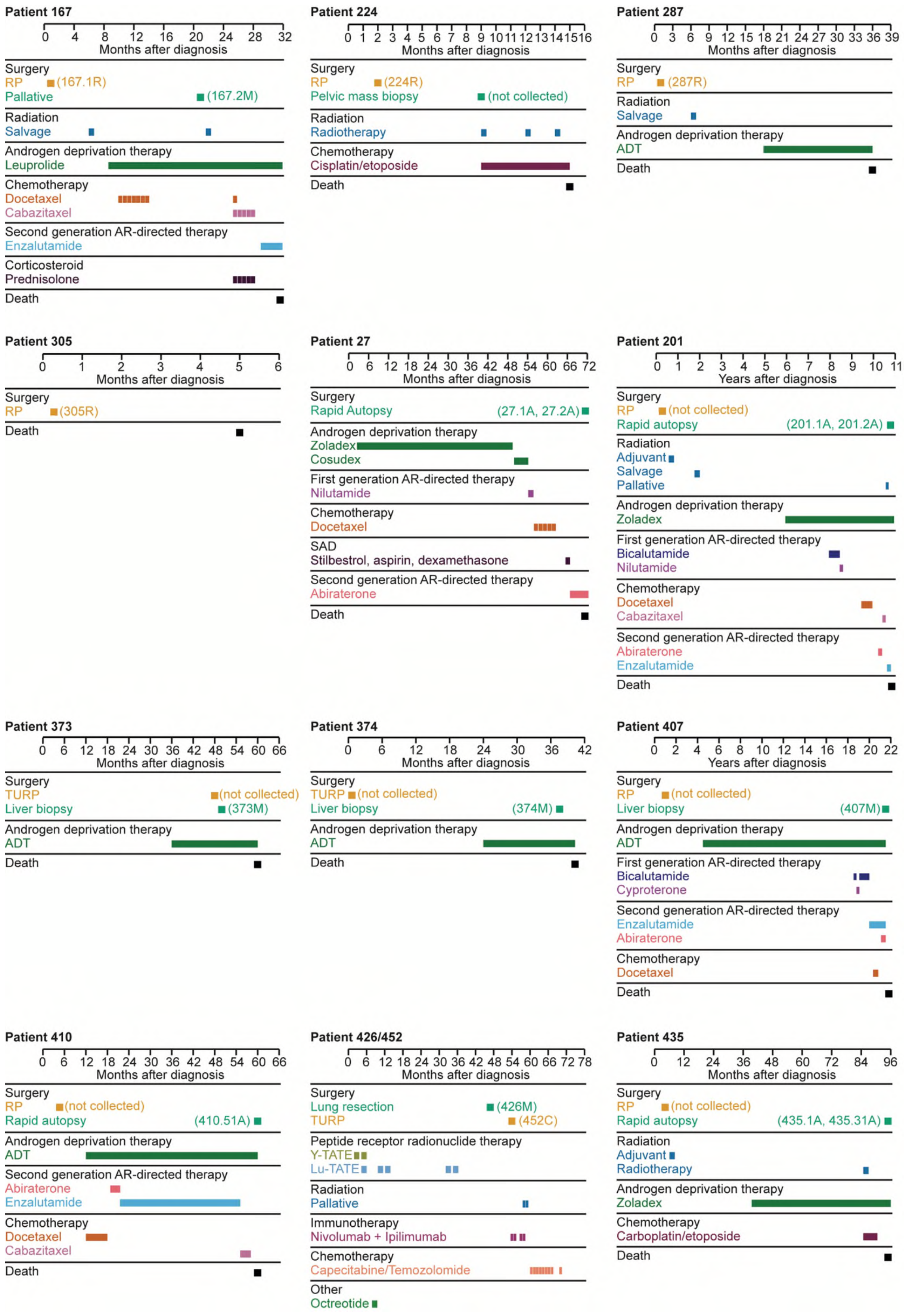

Patient 167
0 4 8 12 16 20 24 28 32
Months after diagnosis
Surgery
RP (167.1R)
Pallative (167.2M)
Radiation
Salvage
Androgen deprivation therapy
Leuprolide
Chemotherapy
Docetaxel
Cabazitaxel
Second generation AR-directed therapy
Enzalutamide
Corticosteroid
Prednisolone
Death
Patient 224
0 1 2 3 4 5 6 7 8 9 10 11 12 13 14 15 16
Months after diagnosis
Surgery
RP (224R)
Pelvic mass biopsy (not collected)
Radiation
Radiotherapy
Chemotherapy
Cisplatin/etoposide
Death
Patient 287
0 3 6 9 12 15 18 21 24 27 30 33 36 39
Months after diagnosis
Surgery
RP (287R)
Radiation
Salvage
Androgen deprivation therapy
ADT
Death
Patient 305
0 1 2 3 4 5 6
Months after diagnosis
Surgery
RP (305R)
Death
Patient 27
0 6 12 18 24 30 36 42 48 54 60 66 72
Months after diagnosis
Surgery
Rapid Autopsy (27.1A, 27.2A)
Androgen deprivation therapy
Zoladex
Cosudex
First generation AR-directed therapy
Nilutamide
Chemotherapy
Docetaxel
SAD
Stilbestrol, aspirin, dexamethasone
Second generation AR-directed therapy
Abiraterone
Death
Patient 201
0 1 2 3 4 5 6 7 8 9 10 11
Years after diagnosis
Surgery
RP (not collected)
Rapid autopsy (201.1A, 201.2A)
Radiation
Adjuvant
Salvage
Pallative
Androgen deprivation therapy
Zoladex
First generation AR-directed therapy
Bicalutamide
Nilutamide
Chemotherapy
Docetaxel
Cabazitaxel
Second generation AR-directed therapy
Abiraterone
Enzalutamide
Death
Patient 373
0 6 12 18 24 30 36 42 48 54 60 66
Months after diagnosis
Surgery
TURP (not collected)
Liver biopsy (373M)
Androgen deprivation therapy
ADT
Death
Patient 374
0 6 12 18 24 30 36 42
Months after diagnosis
Surgery
TURP (not collected)
Liver biopsy (374M)
Androgen deprivation therapy
ADT
Death
Patient 407
0 2 4 6 8 10 12 14 16 18 20 22
Years after diagnosis
Surgery
RP (not collected)
Liver biopsy (407M)
Androgen deprivation therapy
ADT
First generation AR-directed therapy
Bicalutamide
Cyproterone
Second generation AR-directed therapy
Enzalutamide
Abiraterone
Chemotherapy
Docetaxel
Death
Patient 410
0 6 12 18 24 30 36 42 48 54 60 66
Months after diagnosis
Surgery
RP (not collected)
Rapid autopsy (410.51A)
Androgen deprivation therapy
ADT
Second generation AR-directed therapy
Abiraterone
Enzalutamide
Chemotherapy
Docetaxel
Cabazitaxel
Death
Patient 426/452
0 6 12 18 24 30 36 42 48 54 60 66 72 78
Months after diagnosis
Surgery
Lung resection (426M)
TURP (452C)
Peptide receptor radionuclide therapy
Y-TATE
Lu-TATE
Radiation
Pallative
Immunotherapy
Nivolumab + Ipilimumab
Chemotherapy
Capecitabine/Temozolomide
Other
Octreotide
Patient 435
0 12 24 36 48 60 72 84 96
Months after diagnosis
Surgery
RP (not collected)
Rapid autopsy (435.1A, 435.31A)
Radiation
Adjuvant
Radiotherapy
Androgen deprivation therapy
Zoladex
Chemotherapy
Carboplatin/etoposide
Death

Supplementary Fig. 4: Clinical history of patients following tumor collection for research-ready PDXs. Treatment timelines for patients showing each treatment the patient received from diagnosis to current treatment or death. The sample used to establish PDXs is shown in brackets.

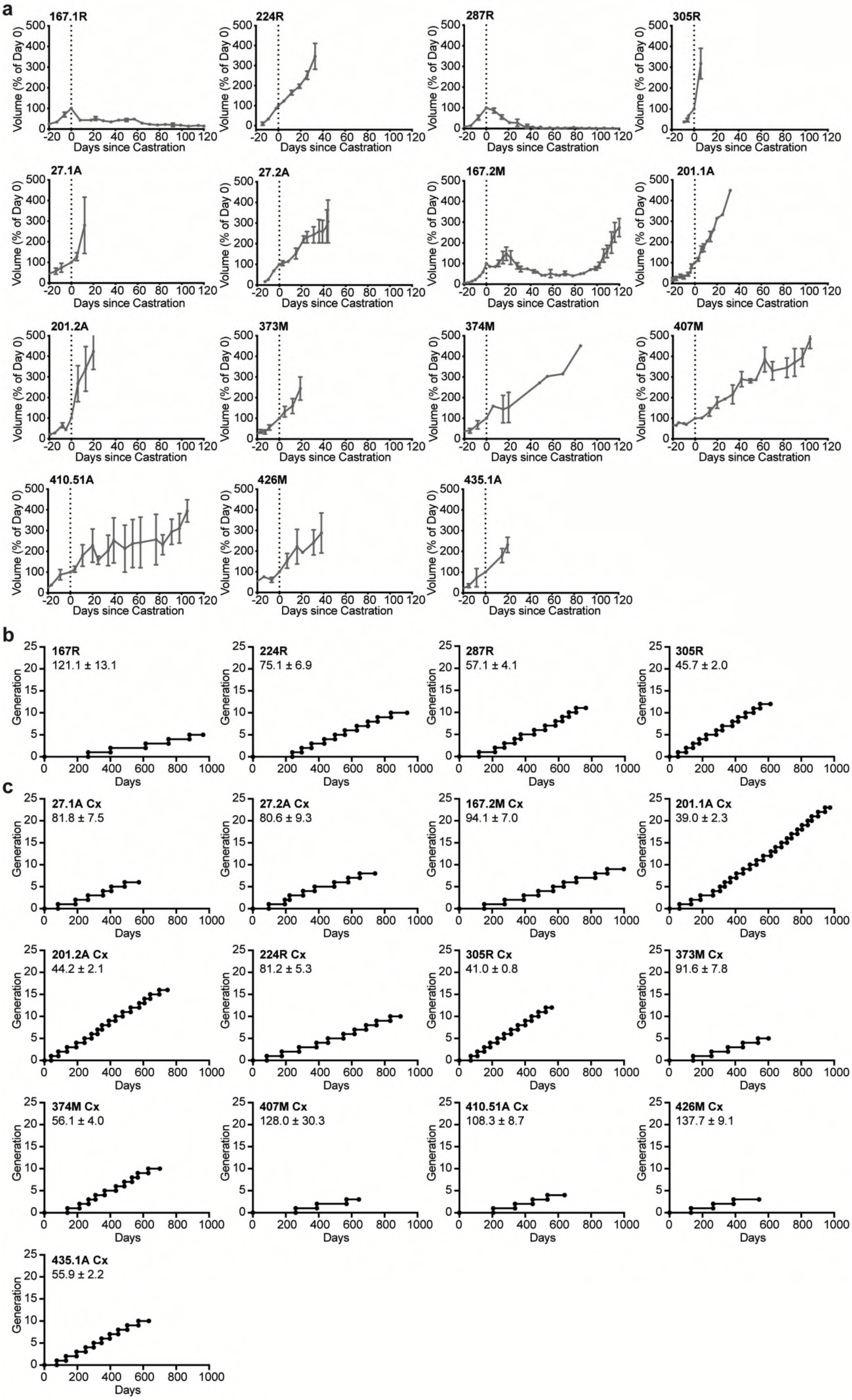

a
167.1R
224R
287R
305R
27.1A
27.2A
167.2M
201.1A
201.2A
373M
374M
407M
410.51A
426M
435.1A
Volume (% of Day 0)
Days since Castration
b
167R
121.1 ± 13.1
224R
75.1 ± 6.9
287R
57.1 ± 4.1
305R
45.7 ± 2.0
c
27.1A Cx
81.8 ± 7.5
27.2A Cx
80.6 ± 9.3
167.2M Cx
94.1 ± 7.0
201.1A Cx
39.0 ± 2.3
201.2A Cx
44.2 ± 2.1
224R Cx
81.2 ± 5.3
305R Cx
41.0 ± 0.8
373M Cx
91.6 ± 7.8
374M Cx
56.1 ± 4.0
407M Cx
128.0 ± 30.3
410.51A Cx
108.3 ± 8.7
426M Cx
137.7 ± 9.1
435.1A Cx
55.9 ± 2.2
Generation
Days

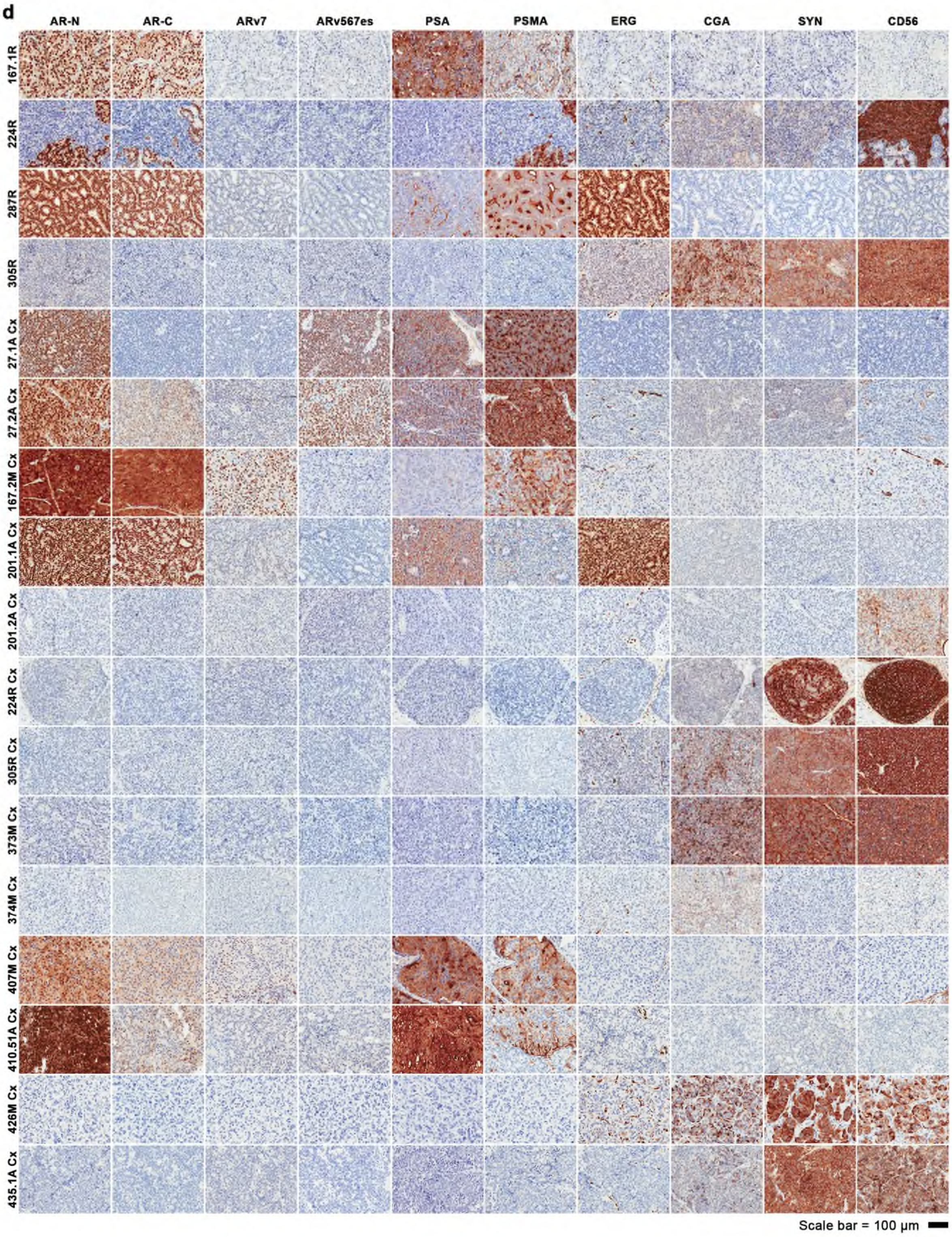
d
AR-N
AR-C
ARv7
ARv567es
PSA
PSMA
ERG
CGA
SYN
CD56
167.1R
224R
287R
305R
27.1A Cx
27.2A Cx
167.2M Cx
201.1A Cx
201.2A Cx
224R Cx
305R Cx
373M Cx
374M Cx
407M Cx
410.51A Cx
426M Cx
435.1A Cx
Scale bar = 100 µm

Supplementary Fig. 5: Characterisation of research-ready PDX collection. (**a**) The response of PDXs to castration. Tumors were established in host mice supplemented with testosterone until tumors reached 200mm^3, at which point host mice were castrated. Graphs show percent change in tumor volume from castration (% Day 0; n = 2 for PDX-305R, -27.2A, -374M and -426M; n = 3 for PDX-167.1R, -224R, -167.2M, -407M and -435.1A; n = 4 for PDX-27.1A, -201.2A, -373M and -410.51A; n = 6 for PDX-287R; n = 8 for PDX-201.1A; data shown as mean ± SEM). (**b-c**). The growth trajectory of PDXs across generations for (**b**) PDXs tumors grown in testosterone-supplemented mice and (**c**) PDXs grown in castrated mice. Each data point represents a different generation with a ≥10-fold increase in tumor volume. Average time per generation is shown, data shown as mean ± SEM. (**d**). The expression of prostate cancer biomarkers in PDXs. Representative images of research-ready PDX collection stained for AR N-terminus (AR-N), AR C-terminus (AR-C), AR-V7, ARv567es, prostate specific antigen (PSA), prostate specific membrane antigen (PSMA), and ERG; and the neuroendocrine markers chromogranin A (CGA), synaptophysin (SYN) and CD56. Scale bar = 100 μm. Staining is repeated every 5th generation across all PDXs. Source data are provided as a Source Data file.

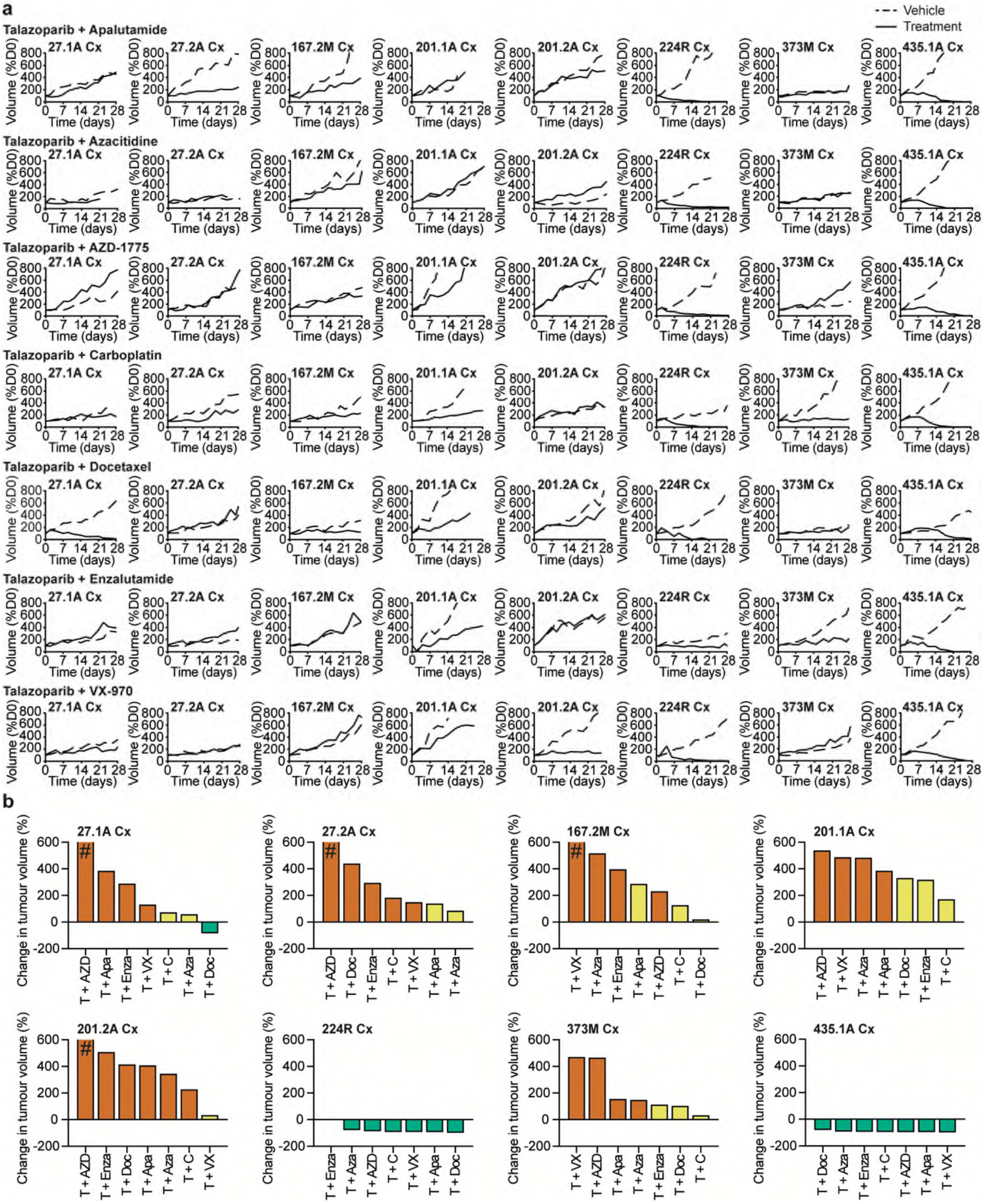
a
Vehicle
Treatment
Talazoparib + Apalutamide
Talazoparib + Azacitidine
Talazoparib + AZD-1775
Talazoparib + Carboplatin
Talazoparib + Docetaxel
Talazoparib + Enzalutamide
Talazoparib + VX-970
27.1A Cx
27.2A Cx
167.2M Cx
201.1A Cx
201.2A Cx
224R Cx
373M Cx
435.1A Cx
Volume (%D0)
Time (days)
b
Change in tumour volume (%)
T + AZD
T + Apa
T + Enza
T + VX
T + C
T + Aza
T + Doc

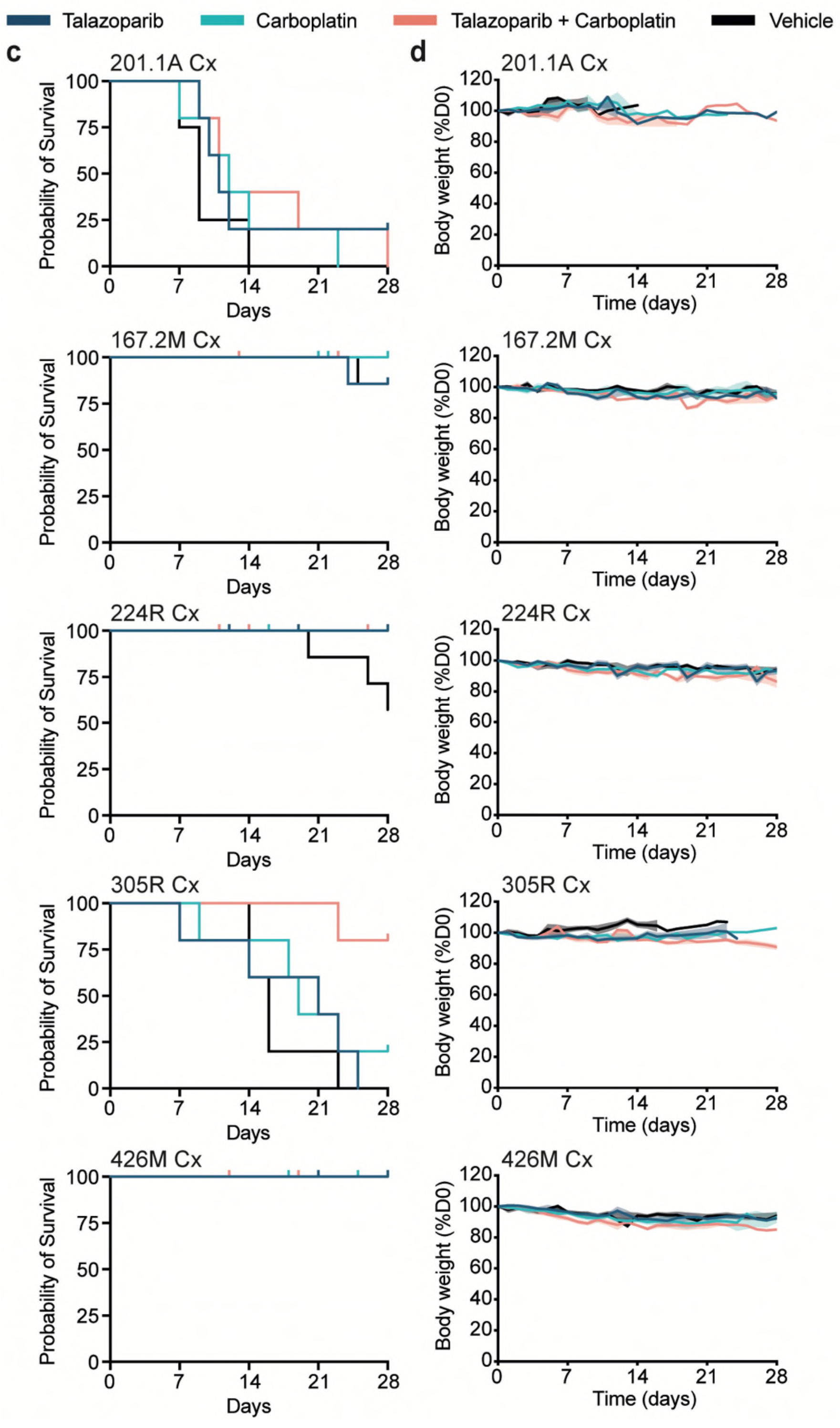

Talazoparib
Carboplatin
Talazoparib + Carboplatin
Vehicle
c
d
201.1A Cx
167.2M Cx
224R Cx
305R Cx
426M Cx
Probability of Survival
Days
Body weight (%D0)
Time (days)

Supplementary Fig. 6: Individual data for PCTs and volcano plots per patient.

(**a**) Graphs show tumor volume (%D0) for PDXs treated with vehicle (dotted line) or combination therapy (solid line) for up to 28 days using the 1x1x1 approach. (**b**) Waterfall plots of the response of to talazoparib combination therapies in eight research-ready PDXs using the one animal per model per treatment (1x1x1) approach after up to 28 days of treatment. Data presented as the percent change in tumor volume compared to day 0 of treatment (%D0), with a response shown in green (tumor volume regressed to <100% of starting volume), a partial response shown in yellow (tumor volume between 100-300% of starting volume and ≤50% volume of matched vehicle) and no response shown in orange (tumor volume >300% of starting volume), # tumor volume increases over 600% are not represented. (**c**) Graphs show Kaplan-Meier survival curves and (**d**) animal body weights for expansion studies treatment of talazoparib and carboplatin combination therapy in five PDXs. Mice were treated for up to 28 days with vehicle (V; black; *n* = 6-8 grafts), 0.33 mg/kg talazoparib (T; dark blue; *n* = 6-8 grafts), 50 mg/kg carboplatin (C; light blue; *n* = 6-8 grafts) or talazoparib and carboplatin (T+C; pink; *n* = 6-8 grafts). Source data are provided as a Source Data file.

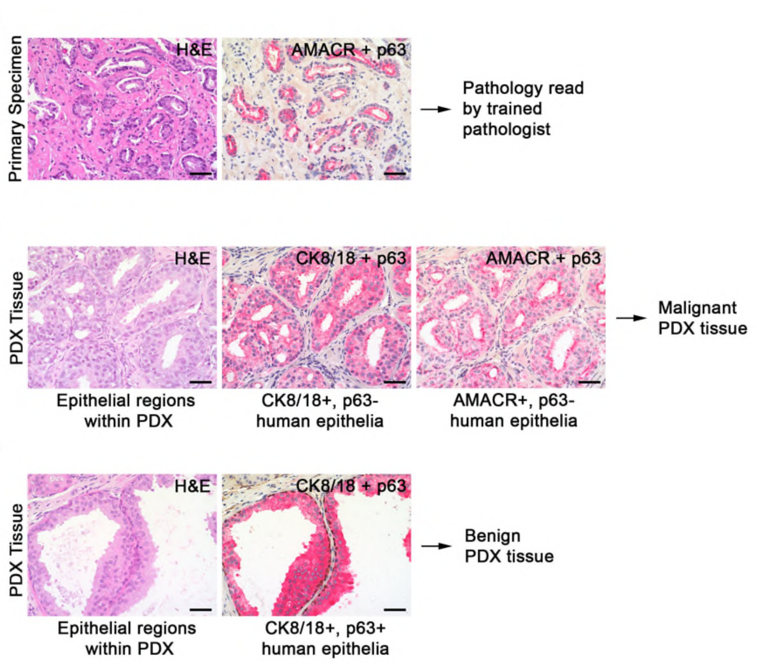

Supplementary Fig. 7: Pathology assessment of original tissues specimens and PDXs. (**a**) Pathological assessment of original tumor tissue was performed by two independent uropathologists using haematoxylin and eosin (H&E) staining and dual immunohistochemistry for the luminal cell marker AMACR (red) and the basal cell marker p63 (brown). (**b-c**) Tumor take rate in PDXs was assessed using H&E staining and immunohistochemistry for human-specific cytokeratin 8/18 (red), AMACR (red) and p63 (brown). PDX tissue which contained CK8/18+/AMACR+/p63- human epithelia was considered malignant (**b**) and PDX tissue which only contained CK8/18+/p63+ human epithelia were considered benign (**c**). Scale bars = 100 µm. Images are representative, pathology assessment is performed on each primary specimen, and every subsequent PDX generation.

Supplementary Tables

Please refer to Supplementary Information

References

1 Gao, D. *et al.* Organoid cultures derived from patients with advanced prostate cancer. *Cell* **159**, 176-187 (2014).

2 Armenia, J. *et al.* The long tail of oncogenic drivers in prostate cancer. *Nat. Genet.* **50**, 645-651 (2018).

3 Robinson, D. *et al.* Integrative clinical genomics of advanced prostate cancer. *Cell* **161**, 1215-1228 (2015).

4 Lawrence, M. G. *et al.* Patient-derived models of abiraterone and enzalutamide-resistant prostate cancer reveal sensitivity to ribosome-directed therapy. *Eur. Urol.* **74**, 562-572 (2018).
