# Supplementary material for: The MURAL collection of prostate cancer patient-derived xenografts enables discovery through preclinical models of uro-oncology
Source: Nat Commun. 2021 Aug 19;12:5049. doi: 10.1038/s41467-021-25175-5 (PMC8376965; doi:10.1038/s41467-021-25175-5)
Supplement: Supplementary file 3 — Description of Additional Supplementary Files [file 41467_2021_25175_MOESM3_ESM.pdf]

Description of Additional Supplementary Files

File Name: Supplementary Data 1
Description: **MURAL prostate cancer PDX collection characteristics**
Table summarising sample site, sample source, patients' clinical features and treatment history, and PDX growth details for each PDX line within the MURAL collection.

File Name: Supplementary Data2
Description: **Prostate cancer biomarker expression in PDXs compared to original patient specimens**
Table showing biomarker expression based on immunohistochemistry in PDXs compared to original patient specimens.

File Name: Supplementary Data 3
Description: **PDX take rate information**
Table summarising the sample site, collection type, tumour survival rate in the first generation PDXs and whether the sample established as a serially transplantable PDX.

File Name: Supplementary Data 4
Description: **Genomics – gene lists and targeted sequencing regions**
Tables listing the genes and targeted sequencing regions included in the Garvan and Twist gene panels for targeting DNA sequencing.

File Name: Supplementary Data 5a
Description: **Genomics – functional alterations – known/likely pathogenic**
Table of targeted sequencing data from PDXs listing single nucleotide variations with known or likely pathogenicity in the curated set of genes that are commonly altered in patient cohorts.

File Name: Supplementary Data 5b
Description: **Genomics – all non-silent SNV and insertions/deletions**
Table of targeted sequencing data from PDXs listing all non-silent single nucleotide variations that were identified.

File Name: Supplementary Data 6a
Description: **Genomics – copy number variants – curated**
Table of targeted sequencing data from PDXs showing a curated set of copy number variants. Copy number calls are relative to the diploid genome and genes are limited to those previously associated with prostate cancer.

File Name: Supplementary Data 6b
Description: **Genomics – all copy number variants**
Table of targeted sequencing data from PDXs showing all copy number variants identified.

File Name: Supplementary Data 7
Description: **Organoid establishment from PDX tissues**
Table showing the establishment of organoids from PDXs, including culture medium, number of passages attempted and organoid growth.

File Name: Supplementary Data 8
Description**: Linear mixed model analyses of tumour volume following treatment with talazoparib and/or carboplatin in PDXs**
Tables of P values comparing graft volume between treatment groups and across time within each treatment group following treatment with vehicle, carboplatin, talazoparib or carboplatin and talazoparib. Statistical analyses were performed using linear mixed model analyses with a test of simple main effects.

File Name: Supplementary Data 9
Description: **Antibody details for Leica BOND-MAXTM automated system**
Table of antibodies and staining conditions for immunohistochemistry.

File Name: Supplementary Data 10
Description: **List of RNA seq PDX samples**
Table showing the PDX line, pathology, sample source and host mouse status for each PDX sample used for RNA sequencing.

File Name: Supplementary Data 11
Description: **Single cell RNA sequencing sample quality and thresholds**
Table showing sample-specific quality control using Cell Ranger and Alevin, filtering thresholds and Seurat parameters for single cell RNA sequencing.
